# Occlusal adjustment using the bite plate-induced occlusal position as a reference position for temporomandibular disorders: a pilot study

**DOI:** 10.1186/1746-160X-6-5

**Published:** 2010-03-27

**Authors:** Kengo Torii, Ichiro Chiwata

**Affiliations:** 1 Torii Dental Clinic, 1-23-2 Ando, Aoi-ku, Shizuoka-shi, 420-0882, Japan; 2 Chiwata Dental Clinic, 2-1-3 Gofukucho, Aoi-ku, Shizuoka-shi, 420-0031, Japan

## Abstract

**Background:**

Many researchers have not accepted the use of occlusal treatments for temporomandibular disorders (TMDs). However, a recent report described a discrepancy between the habitual occlusal position (HOP) and the bite plate-induced occlusal position (BPOP) and discussed the relation of this discrepancy to TMD. Therefore, the treatment outcome of evidence-based occlusal adjustments using the bite plate-induced occlusal position (BPOP) as a muscular reference position should be evaluated in patients with TMD.

**Methods:**

The BPOP was defined as the position at which a patient voluntarily closed his or her mouth while sitting in an upright posture after wearing an anterior flat bite plate for 5 minutes and then removing the plate. Twenty-one patients with TMDs underwent occlusal adjustment using the BPOP. The occlusal adjustments were continued until bilateral occlusal contacts were obtained in the BPOP. The treatment outcomes were evaluated using the subjective dysfunction index (SDI) and the Helkimo Clinical Dysfunction Index (CDI) before and after the occlusal adjustments; the changes in these two indices between the first examination and a one-year follow-up examination were then analyzed. In addition, the difference between the HOP and the BPOP was three-dimensionally measured before and after the treatment.

**Results:**

The percentage of symptom-free patients after treatment was 86% according to the SDI and 76% according to the CDI. The changes in the two indices after treatment were significant (p < 0.001). The changes in the mean HOP-BPOP differences on the x-axis (mediolateral) and the y-axis (anteroposterior) were significant (p < 0.05), whereas the change on the z-axis (superoinferior) was not significant (p > 0.1).

**Conclusion:**

Although the results of the present study should be confirmed in other studies, a randomized clinical trial examining occlusal adjustments using the BPOP as a reference position appears to be warranted.

## Background

Although the role of occlusion in the development of the signs and symptoms of temporomandibular disorder (TMD) remains controversial, occlusal adjustment therapy has been performed for the treatment of TMDs [1-10]. Headaches [11,12], and earaches [13] are often included as symptoms of TMD. However, some current articles have concluded that the available evidence does not support occlusal adjustment as a reasonable therapy for TMDs [14,15]. In addition, the treatment outcome of reversible methods has been proposed as sufficient and appropriate for the management of TMDs, whereas irreversible methods (major alterations in mandibular position or dentoalveolar relationships) do not appear to be necessary for obtaining either short-term or long-term success [16]. TMD is also thought to be related to psychological factors [17]. Nevertheless, many occlusal factors that could be related to the development of TMD have not been thoroughly evaluated.

The use of occlusal adjustments in previous studies [1-12] focused on the correction of a wide range of occlusal conditions (e.g., premature contact in the centric relation (CR), a slide between the intercuspal position (ICP) and the centric relation and occlusal contact on the non-working side) rather than the elimination of a single condition (e.g., only premature contact in the centric relation). Thus, interpreting the results of these treatments is difficult. The components of the treatment must be precisely defined. In previous studies [1-12], the retruded contact position (RCP) was used as the reference position (centric relation) for evaluating occlusion. On the other hand, the muscular position can also be used as a reference position, but this position reportedly varies with the posture of the subject and is thought to be less reproducible than the RCP [18,19]. Therefore, only a few studies examining the effects of occlusal adjustments made using the muscular contact position (MCP) [20] for the treatment of TMD have been published. In more recent studies [21,22], a lightly closed mouth position with the patient in an upright position was used as the MCP and as a reference position; in these studies, the displacement from that position to the clenched position was related to tempromandibular joint (TMJ) noise [21] and the asymmetrical number of occlusal contacts in that position (lightly closed) was related to unilateral TMD symptoms [22]. Most recently, the discrepancy between the habitual occlusal position (HOP) and the flat bite plate-induced occlusal position (BPOP) has been shown to be associated with TMD symptoms [23]. The possible effect of occlusal equilibration in BPOP on TMD symptoms has also been inferred [23].

Therefore, the present study used the BPOP as a reference position and analyzed the occlusion of patients with TMD in relation to this reference position. Evidence-based occlusal adjustment was then performed based on the results of occlusal analysis. After the occlusal adjustment, the outcome was evaluated. In the present study, the HOP obtained by voluntary jaw closing with swallowing while in an upright position was regarded as the mandibular position induced by the jaw motor program of the central nervous system; this position was defined as the intercuspal position (ICP). A voluntary closing position obtained while in an upright position after wearing an anterior bite plate for a short period of time was regarded as the MCP induced by altering the sensory input program and was defined as the BPOP. The present study was performed as a preliminary, open clinical study prior to a randomized clinical trial.

## Materials and methods

Twenty-one patients (three men: mean age, 28.1 years; range, 17-46 years; and 18 women: mean age, 30.2 years; range, 18-66 years) who visited our clinic seeking treatment for TMD were selected for the present study. All the patients provided their informed consent to the BPOP equilibration procedure. The patients were examined and diagnosed for signs and symptoms of TMD using the Research Diagnostic Criteria for TMD (RDC/TMD) [24].

The TMD patients were classified based on the results of clinical examinations as having myofacial pain (5 patients, 24%), disc displacement (14 patients, 67%), or osteoarthritis (2 patients, 9%). Patients with symptoms related to trauma or systemic disease involving joints or muscles were excluded from the study. Patients with a full or nearly full complement of natural teeth, some of which might have been individually restored, were included in the study. Patients with removable prostheses were excluded. None of the patients complained of problems associated with a clear and documentable structural abnormality of the occlusion [15]. None of the patients had received any prior treatment for TMD.

**Figure 1:**
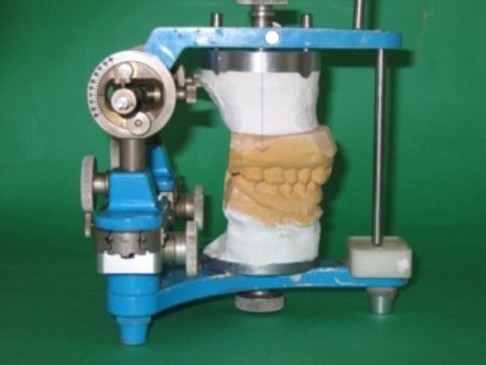
**An articulator used in the study**. After the casts were attached to the articulator, the BPOP wax record was removed from the casts and the upper cast was vertically lowered until the teeth came into contact.

**Figure 2:**
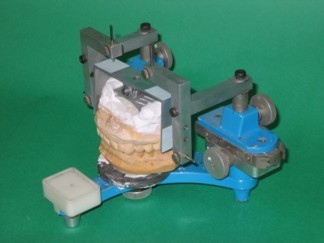
**Mandibular position analyzer**. The apparatus added to an articulator for the three-dimensional analysis of the mandibular position consists of right and left recording arms with pins into the condylar post holes. The recording frame is attached to the upper cast. The mandibular positions are recorded on the frame by the needles on both sides.

One dentist performed the examination and made the diagnosis, and the treatment outcome was evaluated by the same dentist. Another dentist fabricated the anterior bite plate for each patient and performed an occlusal analysis using an articulator with an upper cast vertically movable device (Fig. 1); this dentist also made the occlusal adjustment based on the analysis. Occlusal adjustments were not performed in cases in which premature occlusal contacts existed on the anterior teeth at the BPOP or in which occlusal restorations of the posterior teeth were required. Patients whose teeth would have needed to be ground more than the thickness of the enamel layer for the equilibration of occlusion were also excluded. The occlusal adjustments were performed after the pain and most of the symptoms had subsided as a result of wearing the anterior bite plate. An anterior bite plate was fabricated using self-curing acrylic resin (Ortho-fast; GC, Tokyo, Japan) for each of the patients. The plate covered the upper six anterior teeth and both first premolar teeth. The occlusal surface of the plate was made flat and perpendicular to the mandibular incisors to allow free movement in all directions. Upper and lower dental arch models were made for all the patients. Three occlusal records of the HOP and BPOP were obtained using a vinyl polysiloxane bite registration material (Exabite; GC, Tokyo, Japan) in all the patients while the patient was sitting in an upright position without a head rest for support. The HOP was recorded first, followed by the BPOP. During the recording of the HOP, the patient was asked to swallow and then to close their mouth so as to achieve maximum intercuspation. The patient was then instructed to apply pressure to ensure that the teeth were in contact. This procedure was repeated three times. A vinyl polysiloxane material was applied with a syringe over the occlusal surface. The patient was asked to swallow and then to close their mouth so as to achieve maximum intercuspation, as described previously, and to held that position until the material set (1 minute). To standardize the BPOP recording method, the patient was conditioned neuromuscularly using an anterior bite plate, against which he or she tapped and slid his or her anterior teeth for 5 minutes. After this conditioning, the plate was removed and the patient was asked to close his or her mouth until the point at which his or her teeth came into contact with each other and then to hold that position. This procedure was repeated until the patient could reliably perform the movement. After removing the bite plate, a vinyl polysiloxane material was applied over the occlusal surface and the patient was asked to close his or her mouth in the trained manner, as described previously. Another examiner, who not involved in the recording and was unaware of the patient's status, performed the following measurements and analysis. The discrepancy between the HOP and BPOP was examined using these recordings and the modified articulator. The upper member and condylar posts were replaced with recording arms containing needles, and the recording frame was attached to the upper cast using a specially designed jig. The frame was positioned across both upper first molars. Four graph papers were attached to the horizontal and sagittal surfaces of the recording frame. The trimmed record was interposed between the casts, and different colored occlusal papers were interposed between the graph papers and the recording needles (Fig. 2). The measurements were performed three-dimensionally using a measuring microscope (PIKA measuring microscope PRM-2; PIKA SEIKO Co., Tokyo, Japan) with a resolution of 0.01 mm. The error of this measuring system was ± 0.01 mm for multiple readings by the same examiner for the same record. The errors induced by deviations in the axes and the differences in the width of the dental arches were thought to be negligible.

The difference between the HOP and BPOP was evaluated at the first examination and at a one-year follow-up examination. Before performing an occlusal adjustment, the occlusion was examined using a model attached to the articulator with a BPOP record obtained using a wax registration material (Bite Wafer; Kerr U.S.A., Romulus, MI, U.S.A.) to determine if any premature occlusal contacts existed at the BPOP. The consistency of the BPOP wax records was verified by obtaining three BPOP wax records and using the split cast method.

The purpose of the occlusal adjustment was to obtain occlusal stability in the BPOP. For the occlusal adjustment in the patient's mouth, the anterior bite plate was worn in the mouth; the patient was then asked to tap and slide his or her anterior teeth against the plate for 5 minutes, while in an upright position. The plate was then removed, and the patient was asked to close his or her jaw until tooth contact was made and then to hold that position. Premature contact was located in the mouth by marking and pulling with an occlusal tape (Occlusion foil; Coltene/Whaledent Gmbh Co., Langenau, Germany), and the contact was then removed. The plate was worn again, and the same procedure was repeated until more occlusal contacts on the posterior teeth were obtained. One session of occlusal adjustment was completed within 30 minutes. After completing one session, impressions of both jaws and three BPOP wax records were taken to prepare for the next appointment. The casts were attached to the articulator, and the occlusion was examined using the casts before the next appointment. The occlusal adjustment was completed by confirming the occlusal contacts on the premolar and molar teeth on both sides of the casts attached to the articulator and in the mouth.

### Assessment of subjective symptoms

A questionnaire created by Conti et al. [25] and composed of 10 questions concerning the presence of the most common TMD symptoms was given to each of the patients. For every response indicating the presence of dysfunction, a grade of 2 was given. A score of 0 signified the absence of symptoms, while a grade of 1 corresponded to occasional TMD. A score of 3 was used to indicate severe pain and/or bilateral symptoms. The sum of the scores was used to calculate the subjective dysfunction index (SDI): Di O = no TMD, 0 to 3 points; Di I = mild TMD, 4 to 8 points; Di II = moderate TMD, 9 to 14 points; and Di III = severe TMD, 15 to 21 points. The frequency of headache was classified into four categories: (O) almost never; (I) 1 to 2 times a month; (II) 1 to 2 times a week; and (III) every day. In addition, the graded chronic pain was evaluated as the Axis II profile of RDC/TMD [24]. Scoring for the SCL-R Scales was not performed.

### Assessment of clinical symptoms

The results of a full clinical examination were used to calculate the Helkimo Clinical Dysfunction Index (CDI): Di O = no TMD, 0 points; Di I = mild TMD, 1 to 4 points; Di II = moderate TMD, 5 to 9 points; and Di III = severe TMD, 10 to 25 points [26]. The treatment outcome was evaluated by calculating the SDI and the CDI after treatment and the changes in these indices between the first examination and the one-year follow-up examination; this protocol was implemented to avoid a placebo effect on the treatment outcome and to compare the results in the present study with those from other studies [10,27].

### Statistics

The statistical difference between the HOP and BPOP during the initial and follow-up examinations was analyzed using an analysis of variance for a two-factor experiment with repeated measurements of both position factors. The changes in the statistical difference between the HOP and BPOP before and after treatment were tested using McNemar's test. Differences in subjective and clinical variables before and after treatment were tested using the Wilcoxon's signed-rank test. Spearman's rank correlation coefficient was used to test the significance of any correlation between the changes in subjective and clinical dysfunction scores and confounding factors. The change in the mean difference between the HOP and BPOP before and after occlusal adjustment was tested with a paired t-test.

## Results

**Figure 3:**
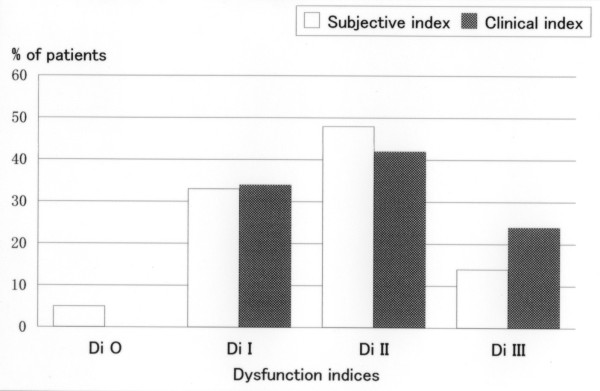
**Dysfunction Index at first examination**. Distribution of dysfunction indices before occlusal adjustment. Di O: no TMD; Di I: mild TMD; Di II: moderate TMD; and Di III: severe TMD.

**Figure 4:**
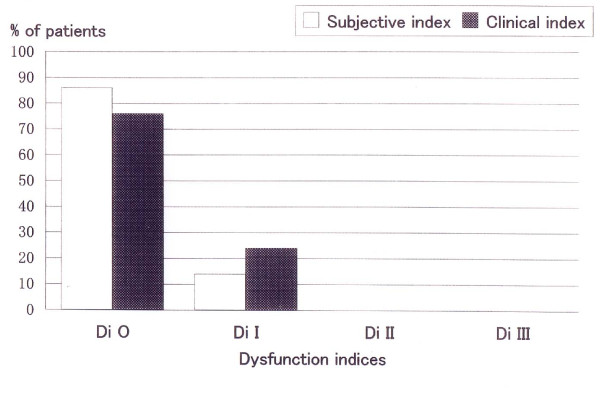
**Dysfunction Index at 1-year evaluation**. Dstribution of dysfunction indices after occlusal adjustment. Di O: no TMD; Di I: mild TMD; Di II: moderate TMD; and Di III: severe TMD.

**Figure 5:**
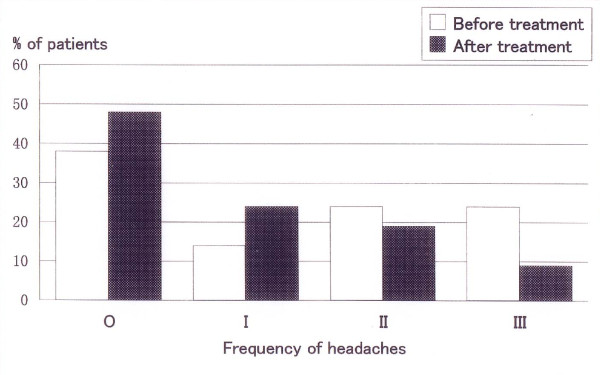
**Headache frequency before and after treatment**. Changes in headache frequency before and after occlusal adjustment. O: almost never; I: 1 to 2 times a month; II: 1 to2 times a week; and III: every day.

**Figure 6:**
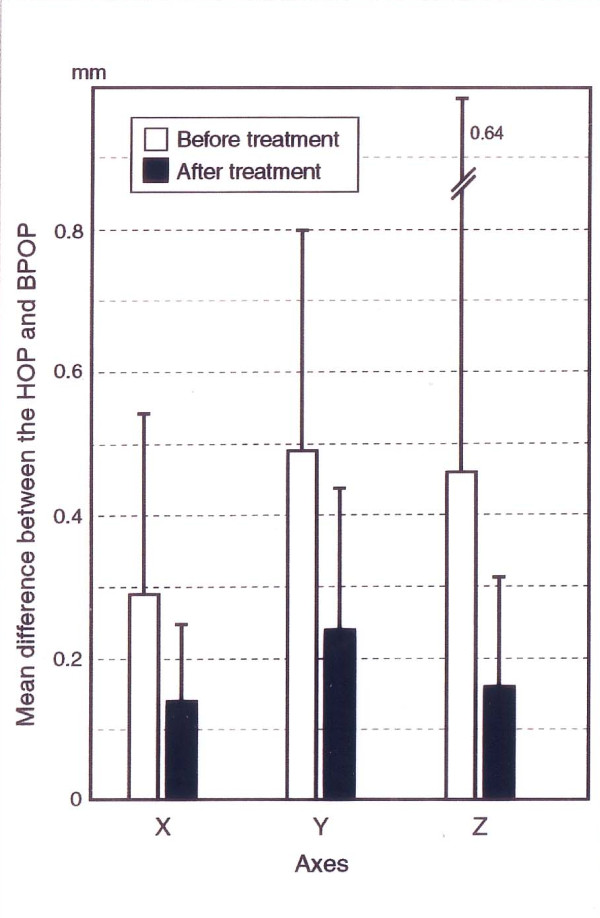
**Mean HOP-BPOP difference before and after treatment**. Changes in the mean difference between the habitual occlusal position (HOP) and the bite plate-induced occlusal position (BPOP) before and after occlusal. adjustment. x: mediolateral; y: anteroposterior; and z:superoinferior.

None of the patients required an anterior bite plate during their one-year follow-up period after the completion of the occlusal adjustment. The number of visits varied between 2 and 34, with a mean of 11.0 ± 6.0 visits. The treatment period ranged between 0.2 and 7.0 months, with a mean of 2.8 ± 2.1 months. The bite plate wearing period ranged from 1 to 21 days, with a mean of 9.6 ± 6.7 days. Between 1 and 13 sessions of occlusal adjustment were performed, with a mean of 4.7 ± 3.5 sessions. The distributions of TMD symptom severity according to the SDI and the CDI indices before and after occlusal adjustment are shown in Fig. 3 and 4, respectively. The SDI had a median value of 9, ranging between 6 and 17 at the time of the first examination, and 1 after the one-year follow-up. The change was statistically significant (p < 0.01). The CDI decreased from a median value of 9 before treatment to 1 after the treatment. This change was statistically significant (p < 0.01). Changes in the frequencies of headaches are shown in Fig. 5. The frequency was significantly lower after occlusal adjustment (p < 0.01). Thirteen patients reported headache symptoms. Five of these patients did not experience any further headaches after treatment. The scores of 8 of the 13 patients with headache symptoms improved by 1 or 2 score categories. The distribution of graded chronic pain on Axis II of RDC/TMD [24] was as follows: grade 0, 3 patients (14%); grade 1, 12 patients (57%); and grade 2, 6 patients (29%) before treatment. After treatment, all the patients had a grade of 0. The changes in the mean difference between the HOP and BPOP before and after occlusal adjustment are shown in Fig. 6. The changes in the mean difference on the x-axis (mediolateral) and y-axis (anteroposterior) were significant (p < 0.05), whereas the change on the z-axis (superoinferior) was not significant (p > 0.1). The changes in the statistical difference between the HOP and BPOP after treatment were statistically significant (Table 1). Confounding factors were not significantly correlated with the changes before and after occlusal adjustment (age: not significant (NS), r_s _= 0.390 for SDI and 0.190 for CDI; times of visit: NS, r_s _= 0.085 for SDI and 0.027 for CDI; treatment period: NS, r_s _= 0.177 for SDI and -0.098 for CDI; and sessions of occlusal adjustment: NS, r_s _= -0.281 for SDI and -0.158 for CDI, respectively). Regarding the subtypes of TMD, the mean numbers of visits were 12.8 ± 6.9 visits for myofacial pain (Myofacial), 10.3 ± 4.9 visits for disc displacement (Disc disp.), and 13.9 ± 8.1 visits for arthritis (Arthritis). The mean lengths of the treatment periods were 3.5 ± 1.2 months for Myofacial, 1.9 ± 1.5 months for Disc disp., and 3.6 ± 2.1 months for Arthritis. The mean numbers of occlusal adjustments were 6.8 times for Myofacial, 4.0 ± 3.1 times for Disc disp., and 4.5 ± 0.7 times for Arthritis. No significant difference in the mean number of visits, the mean lengths of the treatment period, or the mean numbers of occlusal adjustments were observed among the three TMD subtypes when the differences were analyzed using t-tests. Although a meaningful statistical analysis could not be performed because of the small group sizes, the outcomes of the occlusal adjustments for TMD subtypes are shown in Table 2.

**Table 1:** Changes in the statistical difference between the HOP and BPOP before and after occlusal adjustment

**After occlusal adjustment**		
	
**Statistical difference between the HOP and BPOP** **Before occlusal adjustment**	**No difference**	**Difference**
		
Difference	20	0
No difference	1	0

McNemar's test: x^2^_cal _= 18.05>x^2^_1 _(0.001) = 10.83, Significant. HOP: habitual occlusal position; and BPOP: bite plate-induced occlusal position.

**Table 2:** Changes in Helkimo Clinical Dysfunction Index at first examination and after a 1-year follow-up examination for each TMD subtypes

	**Myofacial**		**Disc disp**.		**Arthritis**	
			
**CDI**	**1^st ^Exam**.	**1 y**	**1^st ^Exam**.	**1 y**	**1^st ^Exam**.	**1 y**
Di O	0%	80%	0%	86%	0%	0%
Di I	0%	20%	36%	14%	0%	100%
Di II	40%	0%	43%	0%	50%	0%
Di III	20%	0%	21%	0%	50%	0%

CDI: Helkimo Clinical Dysfunction Index; 1^st ^Exam.: first examination; 1 y: 1-year follow-up examination; Disc disp.: Disc displacement; Myofacial: Myofacial pain. Di O: no TMD; Di I: mild TMD; Di II: moderate TMD; and Di III: severe TMD.

## Discussion

In previous studies, the effects of occlusal adjustment on TMD symptoms have not been confirmed [1-9]. Vallon and Nilner [10] reported that 48% of the patients in their occlusal adjustment group had demanded rescue treatment at the time of a 2-year follow up examination. They concluded that the majority of patients require a comprehensive treatment program, rather than simply an occlusal adjustment. In the present study, none of the patients had demanded rescue treatment at the time of a one-year follow-up examination.

Forssell et al. [14] and Tsukiyama et al. [15] reviewed the published studies on occlusal adjustments and TMD and concluded that no evidence existed to support the use of occlusal adjustments in randomized controlled trials. In previously described studies [1-12], passively guided centric relation was used as the reference position for the procedures of occlusal adjustments. On the other hand, muscular positioning using an actively closing path as a reference position has been proposed. Cooper et al. [20] used a "myocentric" reference position, generated by electrical stimulation of the masticatory muscles and made the HOP consistent with the myocentric position in a patient with myofacial pain dysfunction. However, they did not describe their data in detail. Although they reported a 100% improvement or cure of some symptoms, these results cannot be compared with the results of the present study because of the different kinds of treatment that were employed and the different duration of treatment. In the present study, the BPOP was used as a muscular position for performing occlusal adjustments. This muscular position has been reported to be less reproducible than the RCP or ICP [18,19]. In the present study, a variation in the BPOP was not detected using the split cast method on an articulator or at the occlusal contacts marked in the mouth.

Discrepancies between SDI and CDI improvements have been frequently reported [2,8]. CDI improvements are thought to be of greater importance. The outcomes in the present study were extremely good, as indicated by the large percentages of Di O using both the SDI and CDI grading systems. When the change in the statistical difference between the HOP and BPOP before and after occlusal adjustment in the present study was taken into consideration in addition to the large percentages of Di O in the SDI and CDI systems, the statistical difference between the HOP and BPOP might be inferred to be one of the causes of the TMD. In addition, the difference between the HOP and BPOP in the anteroposterior and mediolateral directions might have some influence on the symptoms of TMD. The continued presence of a premature occlusal contact in the BPOP might cause a discrepancy between the HOP and BPOP, preventing occlusal stability. Such a discrepancy might cause continuous muscular tension when adapting the mandible from the BPOP to the HOP. Under this circumstance, muscular incoordination or muscle pain might be induced. In addition, the displacement from the BPOP to the HOP might distort the proper relationship between the condyle and the TMJ disc and cause TMJ pain or noise [23]. An analysis of occlusion in the BPOP is thought to be important when considering the etiology of TMD-related symptoms. In the present study, all the patients wore an anterior bite plate to relieve their pain and most of their TMD symptoms before the commencement of the occlusal adjustment. Therefore, patients with and without occlusal adjustment after wearing a bite plate should be compared to evaluate the true effects of the occlusal adjustment on the discrepancy between the HOP and BPOP and on the symptoms of TMD.

Regarding headaches, Forssell et al. [11] reported that the treatment of TMD can be an effective therapy for muscle contraction and combination headaches. Karppinen et al. [12] reported that the reduction in headaches was significant in all the patients who had undergone occlusal adjustment, compared with mock-adjustment controls. In the present study, headache frequency was significantly reduced by occlusal adjustment. However, the percentage of patients who experienced a reduction in the frequency of their headaches was smaller than among other patients with other symptoms. Therefore, the mechanism of headache is thought to be more complicated than other TMD symptoms. Considering the good response to occlusal adjustments in the present study, the headaches associated with TMD appear to be tension type headaches with partial involvement of the masticatory muscles. On the other hand, the earaches of one patient and tinnitus of another patient completely disappeared after treatment. Torii and Chiwata reported a case with improved aural symptoms after occlusal equilibration using a BPOP reference position [13].

In the present study, excellent short-term results were obtained using occlusal adjustments made with a BPOP reference position. However, Ommerborn et al. described the importance of psychological factors in general dentistry and in TMD [17]. Tsolka and Preiskel performed a psychological test in their study of occlusal adjustment for TMD [6]. In future studies of occlusal adjustment in BPOP for TMD, psychological testing should be performed. Judging from the results of the TMD subtypes in the present study, the occlusal adjustment using the BPOP should be examined in randomized clinical trials for the TMD subtypes described in the present study.

## Conclusion

Because of the pilot nature of this study, no conclusions can be drawn; however, this initial trial of occlusal adjustment using the BPOP suggests that further study is warranted.

## Competing interests

The authors declare that they have no competing interests.

## Authors' contributions

KT conceived the study, participated in the study's design, and performed the occlusal analysis and selective grinding. IC carried out the diagnosis of TMD, the evaluation of symptoms and the three-dimensional measurements. All authors read and approved the final manuscript.
